# BiLSTM-5mC: A Bidirectional Long Short-Term Memory-Based Approach for Predicting 5-Methylcytosine Sites in Genome-Wide DNA Promoters

**DOI:** 10.3390/molecules26247414

**Published:** 2021-12-07

**Authors:** Xin Cheng, Jun Wang, Qianyue Li, Taigang Liu

**Affiliations:** 1 College of Information Technology, Shanghai Ocean University, Shanghai 201306, China; m200701415@st.shou.edu.cn (X.C.); m200701394@st.shou.edu.cn (Q.L.); 2 School of Software Technology, Zhejiang University, Ningbo 315048, China; sw_junwang@zju.edu.cn

**Keywords:** 5-methylcytosine sites, one-hot encoding, nucleotide property and frequency, bidirectional long short-term memory, majority vote

## Abstract

An important reason of cancer proliferation is the change in DNA methylation patterns, characterized by the localized hypermethylation of the promoters of tumor-suppressor genes together with an overall decrease in the level of 5-methylcytosine (5mC). Therefore, identifying the 5mC sites in the promoters is a critical step towards further understanding the diverse functions of DNA methylation in genetic diseases such as cancers and aging. However, most wet-lab experimental techniques are often time consuming and laborious for detecting 5mC sites. In this study, we proposed a deep learning-based approach, called BiLSTM-5mC, for accurately identifying 5mC sites in genome-wide DNA promoters. First, we randomly divided the negative samples into 11 subsets of equal size, one of which can form the balance subset by combining with the positive samples in the same amount. Then, two types of feature vectors encoded by the one-hot method, and the nucleotide property and frequency (NPF) methods were fed into a bidirectional long short-term memory (BiLSTM) network and a full connection layer to train the 22 submodels. Finally, the outputs of these models were integrated to predict 5mC sites by using the majority vote strategy. Our experimental results demonstrated that BiLSTM-5mC outperformed existing methods based on the same independent dataset.

## 1. Introduction

DNA methylation, which is one of the most studied epigenetic modifications, plays important roles in mammalian development and is associated with a number of key biological processes such as genomic imprinting, repression of transposons and genes, aging, and carcinogenesis [1]. Currently, there are three most common DNA methylation types in living organisms, including N6-methyladenine (6mA), N4-methylcytosine (4mC), and 5-methylcytosine (5mC) [2]. Among them, 5mC is generated when a methyl group is attached to the fifth position of cytosine pyrimidine ring via DNA methyltransferases. In somatic cells, 5mC almost exclusively occurs at palindromic CpG dinucleotides within promoters [3].

An increasing number of human diseases have been found to be associated with aberrant DNA methylation at promoters and regulatory regions [3,4]. For example, many studies have highlighted that the changes in DNA methylation patterns are linked to the initiation of autoimmune rheumatic diseases such as systemic lupus erythematosus [5] and rheumatoid arthritis [6]. Although other epigenetic modifications can also occur in these diseases, DNA methylation is often used as a clinical biomarker and has more practical value due to the stability of the methylated cytosine and its physical association with a specific DNA sequence [7]. In a recent breakthrough, DNA methylation levels can be adopted as biomarkers of aging to estimate the age of any tissue and cell type across the entire life course, forming an accurate epigenetic clock [8]. Subsequently, these biomarkers have been reported to undoubtedly capture pivotal aspects of biological aging and its associated morbidity and mortality [9]. Furthermore, previous research showed that alterations of DNA methylation levels play a critical role in carcinogenesis [10,11]. Genome-wide hypomethylation and the localized hypermethylation of the promoters of tumor-suppressor genes are common epigenetic features of cancer cells and have been recognized as an important component of cancer development and diagnosis [12,13]. Therefore, the accurate identification of 5mC sites in genome-wide DNA promoters is of great importance for understanding the mechanisms and functions of DNA methylation in human genetic diseases such as aging and cancer.

Several traditional high-throughput sequencing techniques, such as bisulfite sequencing [14], oxidative bisulfite sequencing [15], TET-assisted pyridine borane sequencing (TAPS) [16], and Aza-IP [17], have been developed to detect 5mC sites. However, these experimental methods are often time consuming and laborious, insufficient to cope with the explosive growth of nucleotide sequences generated in the post-genomic era [18]. Therefore, it is urgent to explore effective computational methods to identify 5mC sites. To date, various prediction models based on machine learning have been proposed to address this challenge, including Methylator [19], MethCGI [20], iDNA-Methyl [21], and so on. For instance, Bhasin et al. [19] developed a support vector machine (SVM) model called Methylator to identify cytosine methylation in CpG dinucleotides from the MethDB database [22], where every nucleotide was represented by using the conventional binary sparse encoding. Then, Fang et al. explored an SVM-based classifier called MethCGI for predicting methylation status of CpG islands in human brain tissues, using nucleotide sequence contents and transcription factor-binding sites as features [20]. Later, the iDNA-Methyl predictor constructed by Liu et al. achieved remarkable improvements in annotating the DNA methylation sites based on the pseudo-trinucleotide composition and the SVM classifier [21]. Since then, many computational predictors have been proposed to detect 5mC sites in RNA sequences, such as RNAm5Cfinder [23], iRNAm5C-PseDNC [24], RNAm5CPred [25], iRNA-PseTNC [26], m5CPred-SVM [27], iRNAm5C_SVM [28], and so on [29,30,31,32]. For example, Feng et al. designed an SVM-based model to predict 5mC sites in Homo sapiens, in which the RNA samples were encoded using the pseudo-dinucleotide composition [29]. Li et al. explored a web-server named RNAm5Cfinder to identify RNA 5mC sites in eight tissue/cell types from a mouse and human based on the one-hot encoding and the random forest algorithm [23]. Recently, Lv et al. collected experimentally confirmed 5mC data from Homo sapiens, Mus musculus, Saccharomyces cerevisiae, and Arabidopsis thaliana, and developed an optimal predictor called iRNA-m5C for the identification of 5mC sites by comparing the performance of different feature extraction methods and classification algorithms [18]. More reports about the recognition of 5mC sites can be seen in the recent review article [33].

Despite some meaningful achievements that have been made for the detection of DNA/RNA 5mC sites in recent years, there are still two important challenges. First, all previous models were trained on the datasets with the relatively smaller size and have not been applied to predict the 5mC sites in genome-wide DNA promoters. Second, these tools based on machine learning need to take considerable time to extract features and select the optimal feature subset by fusing diverse types of features. To solve these problems, Zhang et al. collected promoter methylation data of the small cell lung cancer (SCLC) from the cancer cell line Encyclopedia (CCLE) database [34,35], and build a deep learning-based predictor called iPromoter-5mC for identifying 5mC modification sites in the promoter region [36]. Based on the one-hot encoding, iPromoter-5mC achieved the robust and reliable performance on the independent testing dataset. Subsequently, Nguyen et al. compared the effectiveness of several of the most popular machine learning techniques including XGBoost, random forest, deep forest, and the deep feedforward neural network based on the same datasets [37]. Among them, the XGBoost classifier with the k-mers embeddings strategy showed the best achievement and outperformed the iPromoter-5mC model on both the 5-fold cross-validation (CV) and the independent test [37].

**Figure 1:**
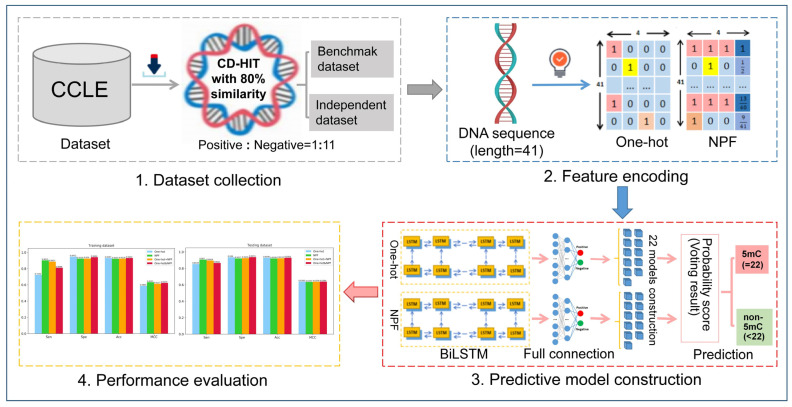
The workflow of the proposed BiLSTM-5mC model for the prediction of 5mC sites.

In this work, we proposed a novel deep learning framework named BiLSTM-5mC for further improving the detection of the 5mC sites in genome-wide DNA promoters. BiLSTM-5mC utilized the one-hot and the nucleotide property and frequency (NPF) methods to encode nucleotide sequences and adopted the bidirectional long short-term memory (BiLSTM) model with a fully connected network to perform the final prediction. To comprehensively evaluate the performance of the proposed model, both the 5-fold CV and the independent test were carried out on the benchmark datasets associated with the SCLC. The experimental results exhibited that BiLSTM-5mC achieved a competitive performance and could be used to help increase predictive levels of DNA 5mC sites in the promoter region from the SCLC. Figure 1 illustrates the framework diagram of the BiLSTM-5mC method.

## 2. Results and Discussions

### 2.1. Sequence Composition Analysis

**Figure 2:**
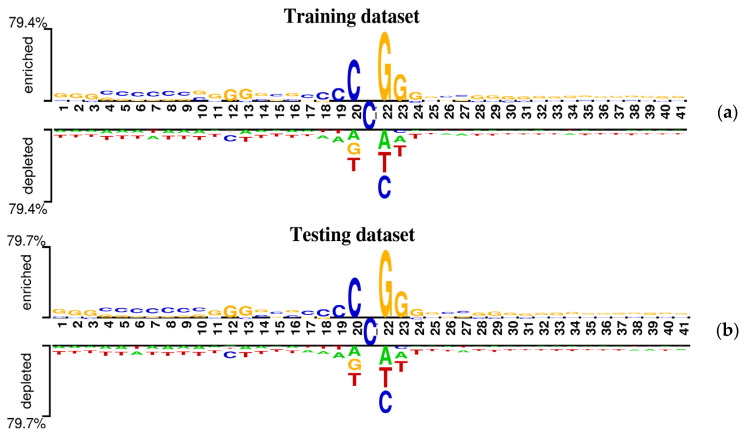
Demonstration of nucleotide composition preferences between positive and negative samples in the benchmark datasets. (**a**) The nucleotide distribution around 5mC sites is for the training dataset. (**b**) The nucleotide distribution around 5mC sites is for the testing dataset.

It is well known that almost all predictors for the identification of 5mC sites are based on the assumption that the sequences around 5mC sites have different nucleotide distributions from the sequences around non-5mC sites. In this study, the sequence context around one potential site can be represented by a sequence window of 41 nucleotides with the modification site at the center. To research the preference of nucleotides distribution around 5mC sites, a web-based tool called Two Sample Logo [38] was employed to statistically analyze the occurrence frequencies of nucleotides at each position between flanking regions of 5mC and non-5mC sites in genome-wide DNA promoters. The statistically significant differences in position-specific nucleotide composition between positive samples (sequences containing 5mC sites) and negative samples (sequences containing non-5mC sites) from the benchmark datasets were graphically represented in Figure 2, in which the nucleotides enriched or depleted in the positive samples are located above or under the horizontal axis, respectively, and the consensus cytosines (C) are displayed in the middle section between the 20 position and 22 position.

As shown in Figure 2, the following observations were obtained. (1) The cytosine (C) and the guanine (G) are prominently enriched in the positive samples and tend to occur at the upstream of 5mC sites. (2) The most conserved motif appears to be “CCGG” at positions 20~23 for the sequence contexts containing 5mC sites. (3) Statistically significant position-specific differences exist between positive and negative samples, indicating the non-random sequence pattern around DNA 5mC sites. Accordingly, it is possible and rational to explore a computational method to predict potential 5mC sites in the promoter region by only using sequence information.

### 2.2. Performance Evaluation on Different Feature Encoding Methods

In this section, we compared the performance of the proposed deep learning framework combined with four different feature encoding schemes, including the one-hot encoding, the NPF strategy, and their concatenation (one-hot+NPF) and combination (one-hot&NPF). As described in Section 3, the one-hot and the NPF methods can encode the nucleotide sequences around 5mC or non-5mC sites into a series of matrices with the size of 41 × 4, respectively. The output of the one-hot+NPF representation is a large matrix of 41 × 8 by concatenating the two matrices with the same size separately generated by the one-hot and the NPF encoding schemes. In contrast, the main idea of the one-hot&NPF strategy is to combinate the outputs of 22 sub-models trained by the proposed deep learning framework, in which 11 sub-models adopt the one-hot encoding and the other 11 sub-models apply the NPF-based features.

**Figure 3:**
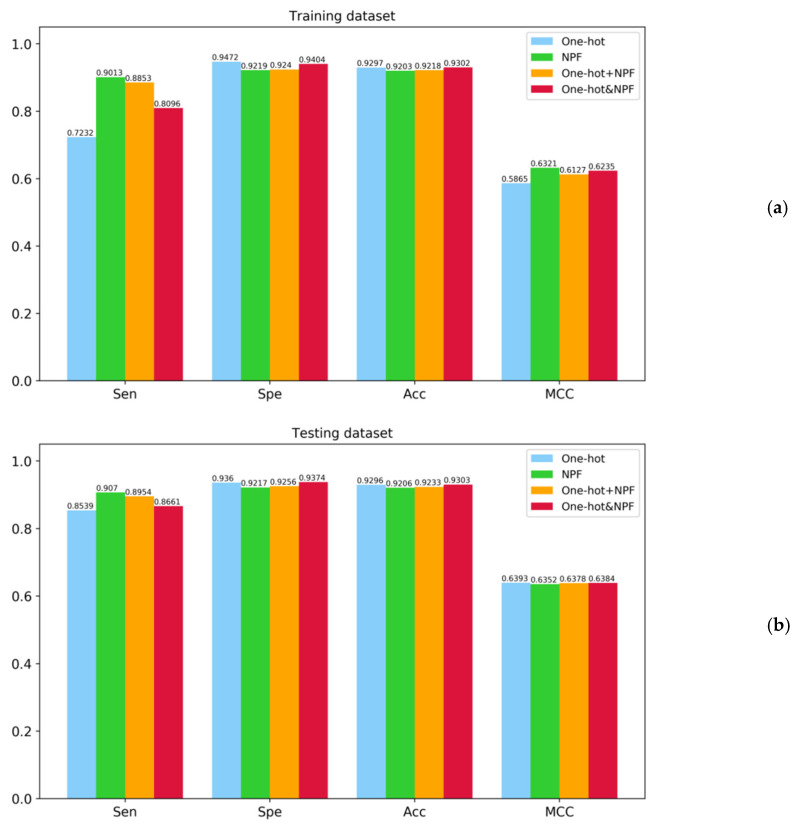
Performance comparison of different feature encoding methods for the prediction of 5mC sites. (**a**) Performance on the training dataset by using the 5-fold CV. (**b**) Performance on the independent testing dataset.

All the results on the training dataset by using the 5-fold CV and on the independent testing dataset were displayed as the histograms visually in Figure 3, where sensitivity (Sen), specificity (Spe), accuracy (Acc), and Matthew’s correlation coefficient (MCC) were selected as the evaluation measures.

**Figure 4:**
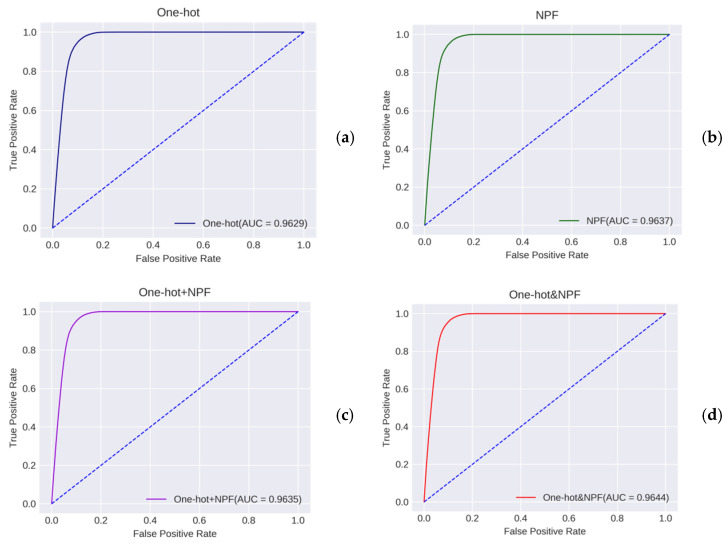
ROC curves of different predictors with different features. (**a**) The ROC curve based on the one-hot features. (**b**) The ROC curve based on the NPF features. (**c**) The ROC curve based on the one-hot+NPF features. (**d**) The ROC curve based on the one-hot&NPF features.

As can be seen from Figure 3a, the proposed BiLSTM-5mC models based on four different feature encoding techniques provided the satisfying performance with the Spe and the Acc higher than 0.92 on the training dataset by using the 5-fold CV. This indicated that the two types of features (i.e., one-hot and NPF) contain position-specific sequence information and could effectively reflect the difference of the nucleotide composition between positive and negative samples. In addition, the model only using the NPF features outperformed the model only using the one-hot encoding in terms of Sen and MCC, maybe due to the sequence-order information encoded in the NPF features. Meanwhile, the ensemble predictor with the one-hot&NPF encoding was superior to the predictor with the one-hot+NPF encoding in terms of Spe (0.9404), Acc (0.9302), and MCC (0.6235). Similar conclusions could be reached from Figure 3b. For the independent testing dataset, the combination of the BiLSTM model and the one-hot&NPF representation exhibited the highest Spe (0.9374) and Acc (0.9303) and obtained the acceptable Sen value (0.8661) and MCC value (0.6384). This suggested that the proposed deep learning framework could serve as a powerful tool for the recognition of 5mC sites. Additionally, the receiver operating characteristic (ROC) curves associated with these models were plotted in Figure 4, which demonstrated the similar conclusions as Figure 3. The area under the ROC curve (AUC) values of these predictors were higher than 0.96.

### 2.3. Performance Comparison with Existing Methods

To the best of our knowledge, there are only two computational tools to identify 5mC sites in genome-wide DNA promoters on the same datasets, i.e., iPromoter-5mC [36] and 5mC_Pred [37]. Table 1 provides an overview of these tools and our model, including feature description methods and classification algorithms. For a fair comparison with existing methods, we adopted the same training dataset and independent testing dataset to objectively evaluate the identification performance. The corresponding comparison results are reported in Table 2 and Table 3 using the following five common metrics: Sen, Spe, Acc, MCC, and AUC.

As illustrated in Table 2, the proposed BiLSTM-5mC model attained the best performance in terms of Spe (0.9404), Acc (0.9302), and AUC (0.9644). However, the Sen value of our method was lower than those of other predictors. This may be caused by the extreme imbalance between the number of positive and negative samples in the dataset. In addition, 5mC_Pred provided an outstanding performance with the Sen value close to 0.9 and the highest MCC value, which adopted the FastText algorithm to generate embedding vectors [37]. This indicated that k-mers embeddings learned from a pre-trained language model could improve the capability of the model to discriminate 5mC sites from non-5mC sites. Referring to Table 3, BiLSTM-5mC showed the acceptable Sen value and the best predictive power in terms of Spe (0.9374), Acc (0.9303), MCC (0.6384), and AUC (0.9635) compared to iPromoter-5mC and 5mC-Pred. The possible cause was that the BiLSTM model could make up for the lack of time information in the one-hot encoding and capture the long-range information of DNA sequences.

In summary, BiLSTM-5mC achieved an excellent performance and outperformed the other existing tools both on the training dataset and the independent testing dataset. These comparison results demonstrated that BiLSTM-5mC is a powerful predictor with the capability of accurately predicting the potential 5mC sites. We hope that our approach might be effectively used for the large-scale annotation of 5mC sites.

## 3. Materials and Methods

### 3.1. Benchmark Datasets

The construction of a high-quality benchmark datasets is the prerequisite step in developing a robust and reliable classification model. In the present work, the benchmark datasets constructed by Zhang et al. [36] were directly applied to train and test our proposed method, including 69,750 positive and 823,576 negative 5mC samples. Specifically, all the samples in this dataset were nucleotide sequences with the length of 41 and the cytosine at the center, which were collected from the entire genome in the SCLC of the CCLE database [34,35]. In order to reduce the homology bias, the sequences that had more than 80% sequence similarity with any other sequences were removed by using the CD-HIT software [39]. Using the same strategy in that work [36], the total samples were randomly divided into the training dataset and the independent testing dataset. As a result, the training dataset contained 55,800 5mC samples and 658,861 non-5mC samples, while the remaining 13,950 5mC samples and 164,715 non-5mC samples were adopted as the independent testing dataset. Although the proportion of positive samples and negative samples was about 1:11, this unbalanced data could more objectively reflect the distribution of 5mC modification sites in the promoter region. The details of the benchmark datasets are presented in Table 4.

### 3.2. Feature Encoding Schemes

#### 3.2.1. One-Hot Encoding

The one-hot encoding is a simple and effective feature representation scheme for the classification of the DNA sequences, which can describe the nucleotide composition along the DNA sequences. For this encoding scheme, four types of nucleotides, namely adenine (A), cytosine (C), guanine (G), and thymine (T), are represented as (1, 0, 0, 0), (0, 1, 0, 0), (0, 0, 1, 0), and (0, 0, 0, 1), respectively. Accordingly, the DNA sample with the length of 41 in the dataset is encoded into a 41 × 4-dimensional vector.

#### 3.2.2. The Nucleotide Property and Frequency

The nucleotide is the basic structural and functional unit of DNA, and its chemical property can impact the inherited characteristics of DNA sequences to some degree. In addition, the cumulative frequency characteristics of nucleotide in the DNA sequence can capture the sequence-order and position-specific information. Similar to the one-hot encoding scheme, the NPF features have been widely used for the computational identification of DNA or RNA modification sites [40,41,42].

According to the NPF method, the i-th nucleotide $n_{i}$ (1≤i≤41) in the DNA sequence can be represented by a four-dimensional vector $\left(x_{i},y_{i},z_{i},d_{i}\right)$. These elements are defined as follows:

(1) $$x_{i}=\{\begin{array}{c} 1,\;\text{if}\;n_{i}\in \left\{A,G\right\}\\ 0,\;\text{otherwise } \end{array}$$

(2) $$y_{i}=\{\begin{array}{c} 1,\;\text{if}\;n_{i}\in \left\{A,C\right\}\\ 0,\;\text{otherwise} \end{array}$$

(3) $$z_{i}=\{\begin{array}{c} 1,\;\text{if}\;n_{i}\in \left\{A,T\right\}\\ 0,\;\text{otherwise} \end{array}$$

(4) $$d_{i}=\frac{1}{\left|N_{i}\right|}\overset{\left|N_{i}\right|}{\underset{j=1}{\sum }}f\left(n_{j}\right)$$

where

(5) $$f(n_{j})=\{\begin{array}{c} 1,\;\text{if}\;n_{j}=n_{i}\\ 0,\;\text{otherwise} \end{array},\;\;\;n_{i},n_{j}\epsilon \left\{A,C,G,T\right\},$$

where $\left|N_{i}\right|$ is the length of the prefix string from the first position to the position i of the sequence, $d_{i}$ is the accumulated frequency of the nucleotide $n_{i}$ in the prefix string, and the first three elements (i.e., $x_{i},y_{i},z_{i})$ stand for the ring structure, chemical functionality, and hydrogen bond of the nucleotide $n_{i}$, respectively. As a result, each query 5mC sample is converted into a 41 × 4-dimensional vector by using the NPF encoding.

### 3.3. Model Construction

#### 3.3.1. The Overall Framework

**Figure 5:**
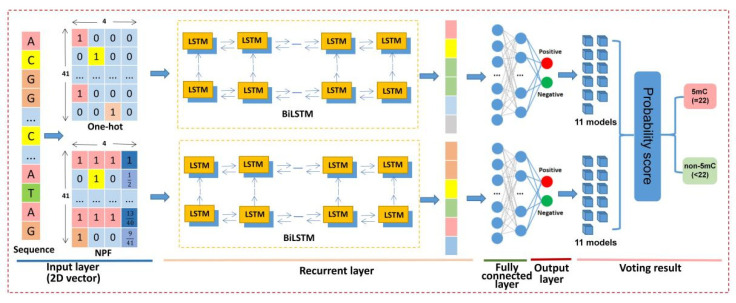
The structure of the BiLSTM-5mC framework.

To solve the imbalance problem between positive samples and negative samples, the under-sampling method was adopted in this study. Firstly, we randomly divided the negative samples from the training dataset into 11 groups with the equal size, one of which can be combined with the same amount of the positive samples to form the balanced training subset. Next, a query sequence was converted into numerical vectors with the fixed length by using the one-hot and NPF encoding. Then, these vectors were input into 22 sub-models acquired by the BiLSTM model and the fully connected network for predicting the category of the query sequence. Finally, the 22 predictive results were integrated to determine whether the query sequence was a 5mC sample or not by using a simple majority voting method. The above comprehensive predictor was named as BiLSTM-5mC, whose overall framework was illustrated in Figure 5.

#### 3.3.2. Bidirectional Long Short-Term Memory Network

DNA sequence analysis is similar to natural language processing, in which recurrent neural networks (RNNs) can be used to process the sequential data. As a popular and powerful RNN architecture, LSTM has been widely used to solve the problem of biological sequence analysis and has achieved excellent performance [43,44,45]. BiLSTM consists of two reversed unidirectional LSTM networks, which is a special type of RNN. BiLSTM can integrate both forward and backward information in a sequence and capture the mutual dependence across the sequence [46]. In BiLSTM-5mC, two BiLSTM models were designed to process the one-hot and NPF feature encoding schemes. Subsequently, the apiece feature matrix generated by the apiece encoding scheme was converted into a one-dimensional feature vector as the input of the full connection layer to perform the classification.

#### 3.3.3. Fully Connected Network

We utilized the one-hot and NPF features as the input to train the BiLSTM models in parallel. Then, the resulting feature vectors were fed into a fully connected network with three layers. Both the first and second layer of the fully connected network contained 300 neurons, and the last output layer contained two units for predicting two classes (i.e., 5mC sample and non-5mC sample). Additionally, sigmoid was selected as the activation function.

When the voting strategy was applied to integrate all the decisions originated from the 22 sub-models into the final assignment result, a strict identification standard was utilized. Specifically, if only all the sub-models judged that the query sample is a true 5mC modification site, the BiLSTM-5mC model could decide that the center nucleotide of the query sequence is a 5mC site.

### 3.4. Performance Evaluation

To rigorously and impartially measure the performance of the proposed model, we implemented the 5-fold CV and the independent dataset test based on the benchmark datasets. In this work, the five common evaluation metrics were reported, including sensitivity (Sen), specificity (Spe), accuracy (Acc), and the Matthews correlation coefficient (MCC). They are defined as follows:

(6) $$\mathrm{Sen}=\frac{TP}{TP+FN}\;,$$

(7) $$\mathrm{Spe}=\frac{TN}{TN+FP}\;,$$

(8) $$\mathrm{Acc}=\frac{TP+TN}{TP+TN+FP+FN}\;,$$

(9) $$\mathrm{MCC}=\frac{TP\times TN-FP\times FN}{\sqrt{\left(TP+FP\right)\times \left(TP+FN\right)\times \left(TN+FP\right)\times \left(TN+FN\right)}}\;,$$

where *TN*, *TP*, *FN*, and *FP* denote the numbers of the true negative, true positive, false negative, and false positive, respectively. Moreover, for the sake of illustrating the diagnostic ability of our predictor visually, the receiver operating characteristic (ROC) curve emerged by plotting the true positive rate (Sen) against the false positive rate (1-Spe) at different thresholds. The area under the ROC curve (AUC) was also calculated as a powerful performance metric and provided in the ROC figure.

## 4. Conclusions

In this study, we proposed a deep learning-based approach, called BiLSTM-5mC, for accurately identifying 5mC sites in genome-wide DNA promoters in cell lines of the SCLC. The main innovative points of our model existed in the following two aspects. First, we adopted the under-sampling method to solve the imbalance problem between positive samples and negative samples. The overabundant negative samples were randomly divided into 11 groups, one of which had an approximately equal size with the positive samples. Second, the deep learning frame based on the BiLSTM model and the fully connected network was explored to perform the identification of 5mC sites by capturing the sequence-order and position-specific information from the one-hot and NPF features. Benchmarking experiments demonstrated that the proposed BiLSTM-5mC model exhibits a competitive performance compared with two existing algorithms and could serve as a useful tool for helping increase the annotation levels of 5mC sites.

## Figures and Tables

**Table 1:** Summary of existing tools for 5mC sites prediction in genome-wide DNA promoters.

Method	Feature	Algorithm
iPromoter-5mC [36]	One-hot	Deep neural network
5mC_Pred [37]	K-mers	XGBoost
BiLSTM-5mC (This study)	One-hot and NPF	BiLSTM

**Table 2:** Performance comparison on the training dataset by using the 5-fold CV.

Method	Sen	Spe	Acc	MCC	AUC
iPromoter-5mC	0.8746	0.9039	0.9016	0.5743	0.9566
5mC_Pred	0.8990	0.9200	0.9180	0.6260	0.9620
BiLSTM-5mC	0.8096	0.9404	0.9302	0.6235	0.9644

**Table 3:** Performance comparison on the independent testing dataset.

Method	Sen	Spe	Acc	MCC	AUC
iPromoter-5mC	0.8777	0.9042	0.9022	0.5771	0.9570
5mC_Pred	0.8950	0.9200	0.9180	0.6250	0.9620
BiLSTM-5mC	0.8661	0.9374	0.9303	0.6384	0.9635

**Table 4:** The information of the experimental datasets.

Dataset	Positive Sample	Negative Sample
Training dataset	55,800	658,861
Testing dataset	13,950	164,715
Total	69,750	823,576

## Data Availability

The data and the source code used to support the findings of this study are freely available to the academic community at https://github.com/taigangliu/BiLSTM-5mC, accessed on 5 December 2021.

## Sample Availability

Samples of the compounds are not available from the authors.
